# Metabolic dysfunction-associated fatty liver disease increases the risk of new-onset type 2 diabetes mellitus: a retrospective cohort study based on health check-up data

**DOI:** 10.3389/fmed.2026.1881173

**Published:** 2026-09-16

**Authors:** Lihua Sun, Feng Chen, Jing Li

**Affiliations:** Health Management Center, Shanxi Bethune Hospital (Shanxi Academy of Medical Sciences Tongji Shanxi Hospital), Third Hospital of Shanxi Medical University, Taiyuan, China

**Keywords:** cohort study, health check-up, metabolic dysfunction-associated fatty liver disease, risk factor, type 2 diabetes mellitus

## Abstract

**Background:**

Metabolic dysfunction-associated fatty liver disease (MAFLD) is the most prevalent chronic liver condition globally, yet its prospective association with incident type 2 diabetes mellitus (T2DM) in the general health check-up population requires further elucidation. This study aimed to investigate whether baseline MAFLD increases the risk of new-onset T2DM in a health check-up cohort.

**Materials and methods:**

This retrospective cohort study initially screened 1,520 individuals from the health check-up database; after applying strict exclusion criteria (including baseline T2DM, other liver diseases, malignancy, missing key data, and loss to follow-up), 186 participants without baseline T2DM were finally included. These participants underwent annual health examinations at Shanxi Bethune Hospital Health Check-up Center from 2020 to 2024. Baseline anthropometric measurements, biochemical parameters, and medical histories were collected. The primary outcome was new-onset T2DM over 5 years. Associations were analyzed using Kaplan–Meier methods and multivariable Cox regression. The primary analysis was adjusted for age, sex, and body mass index (BMI); a fully adjusted model including additional metabolic covariates was performed as a sensitivity analysis.

**Results:**

Over a median 60-month follow-up, 22 new T2DM cases occurred. The 5-year cumulative incidence of T2DM was significantly higher in the MAFLD group than in the non-MAFLD group (18.9% vs. 5.6%, *p* = 0.001). Multivariable Cox regression suggested that MAFLD was associated with an increased risk of new-onset T2DM (adjusted HR = 3.15, 95%CI: 1.58–6.28, *p* = 0.001 for age, sex, and BMI; a fully adjusted sensitivity analysis yielded HR = 2.71, 95%CI: 1.33–5.52, *p* = 0.006), with preliminary findings for both lean MAFLD (HR = 2.88, 95%CI: 1.10–7.54, based on a small subgroup of 15 participants) and overweight/obese MAFLD (HR = 2.65, 95%CI: 1.22–5.76, both from fully adjusted sensitivity models). Sensitivity and stratified analyses were generally consistent with the primary association, although the small number of events in subgroup analyses limited the statistical power and precluded definitive conclusions.

**Conclusion:**

Baseline MAFLD may be associated with an increased risk of new-onset T2DM within 5 years in a health check-up population, although these preliminary findings require confirmation in larger studies. Both lean and overweight/obese MAFLD subtypes showed associations with an elevated risk of T2DM in exploratory analyses, although the finding for lean MAFLD (based on only 15 individuals and 3 T2DM events) should be considered hypothesis-generating and preliminary and requires validation in larger cohorts. Early screening and lifestyle interventions for individuals with MAFLD are of paramount clinical importance for diabetes prevention.

## 1 Introduction

Type 2 diabetes mellitus (T2DM) is a rapidly growing global health challenge, with prevalence projected to exceed 700 million by 2045, placing a substantial burden on healthcare systems worldwide (1). In China, which has the largest diabetic population globally, the age-standardized prevalence of T2DM among adults is 12.8%, highlighting the urgent need for early identification of high-risk individuals and preventive interventions (2). T2DM pathogenesis involves genetic and environmental factors, with insulin resistance and beta-cell dysfunction as core hallmarks (3). Identifying modifiable risk factors and intermediate phenotypes preceding overt hyperglycemia is therefore critical to curbing the diabetes epidemic.

Metabolic dysfunction-associated fatty liver disease (MAFLD) affects an estimated 30% of the global adult population (4). Introduced in 2020, the MAFLD nomenclature emphasizes metabolic dysfunction as the core pathogenesis, with diagnosis requiring hepatic steatosis plus overweight/obesity, type 2 diabetes, or at least two metabolic abnormalities (5). This redefinition better aligns with the underlying pathophysiology and has key implications for understanding its systemic consequences, particularly cardiometabolic disorders (6). In 2023, a multi-society Delphi consensus proposed a nomenclature change from MAFLD to metabolic dysfunction-associated steatotic liver disease (MASLD), with modified diagnostic criteria (6). While the present study was designed and initiated prior to the widespread adoption of MASLD terminology, and we used the MAFLD criteria as defined in 2020 for internal consistency throughout the study, we acknowledge that MAFLD and MASLD are related but not identical constructs. The MAFLD definition requires the presence of hepatic steatosis plus overweight/obesity, T2DM, or at least two metabolic abnormalities, whereas MASLD requires hepatic steatosis plus at least one of five cardiometabolic risk factors in the absence of other causes of liver disease. This difference may affect the composition of the exposed population and thus the comparability of our findings with studies using the MASLD criteria. We have therefore elected to use MAFLD terminology throughout to maintain consistency with our exposure definition and to facilitate comparison with the substantial body of literature that has used the MAFLD criteria.

The association between fatty liver disease and T2DM is well established, underpinned by shared insulin resistance (7). The liver is central to glucose homeostasis, and intrahepatic fat accumulation drives hepatic insulin resistance, increasing gluconeogenesis and reducing glycogen synthesis (8). Meta-analyses show that NAFLD confers an approximately two-fold increased risk of T2DM (9). However, most studies used the prior NAFLD definition-lacking metabolic specificity-and were hospital-based, introducing selection bias (10). In addition, steatosis was often assessed by semi-quantitative ultrasound, and follow-up duration and adjustment for metabolic confounders were often suboptimal (11).

Several critical gaps persist in the literature, particularly in the context of the newly established MAFLD definition. First, large-scale, population-based cohort studies utilizing the MAFLD criteria, especially within the Chinese health check-up population, remain scarce (12). Second, it remains controversial whether distinct MAFLD subtypes, such as lean MAFLD (individuals with hepatic steatosis despite a normal body mass index), confer differential risks for incident T2DM (13). Third, inadequate control for potential confounding factors, such as detailed lipid profiles and liver enzymes, may have led to overestimation or underestimation of the true association in previous studies (14). Additionally, the prognostic value of MAFLD for T2DM in the context of routine health screening, where asymptomatic individuals undergo periodic evaluations, has not been sufficiently explored (15).

Therefore, this retrospective cohort study was designed to leverage five-year longitudinal data from a health check-up center in China to address these gaps. The primary objectives were to evaluate the association between baseline MAFLD and the risk of new-onset T2DM, to explore whether the risk varies across different MAFLD subtypes (lean vs. overweight/obese), and to validate the robustness of our findings through a series of stratified and sensitivity analyses. The findings from this study may provide preliminary, contemporary, real-world evidence suggesting that MAFLD could be considered a potential risk marker for T2DM in routine health management, although further validation in larger cohorts is warranted.

## 2 Materials and methods

### 2.1 Study design and ethical approval

This single-center, retrospective cohort study used electronic health records from the Shanxi Bethune Hospital Health Check-up Center. The Institutional Review Board of Shanxi Bethune Hospital Institution approved the protocol, and informed consent was waived due to the retrospective, de-identified nature of the data.

### 2.2 Study population

We screened the health check-up records of all adults (age ≥18 years) who underwent a comprehensive annual health examination at the Shanxi Bethune Hospital Health Check-up Center between January 1, 2020, and December 31, 2020 (baseline visit). All participants were required to have completed at least one follow-up health examination by December 31, 2024. Inclusion criteria were age ≥18 years, completion of baseline health examination in 2020, completion of at least one follow-up examination by 2024, and absence of T2DM at baseline. Exclusion criteria were presence of T2DM at baseline (defined as fasting plasma glucose ≥7.0 mmol/L, self-reported history of physician-diagnosed T2DM, or use of glucose-lowering medications), presence of other liver diseases including viral hepatitis (positive hepatitis B surface antigen or anti-hepatitis C virus antibody), alcoholic liver disease, drug-induced liver injury, or autoimmune hepatitis, history of any malignancy, severe hepatic or renal insufficiency, and missing key baseline data (such as liver ultrasound or fasting plasma glucose) or loss to follow-up during the 5-year study period.

To assess the potential for selection bias, we compared the baseline characteristics between the 186 included participants and the 1,334 excluded individuals (including those with baseline T2DM, other liver diseases, malignancy, missing key data, or loss to follow-up) using available data from the initial screening visit. This comparison is presented in Supplementary Table S1. Briefly, included participants were younger (46.5 ± 10.9 vs. 51.2 ± 12.3 years, *p* < 0.001), had lower BMI (24.9 ± 3.2 vs. 26.1 ± 3.5 kg/m^2^, *p* < 0.001), lower fasting glucose (5.35 ± 0.48 vs. 5.58 ± 0.62 mmol/L, *p* < 0.001), and lower ALT levels (26.0 [19.0–35.0] vs. 30.0 [22.0–42.0] U/L, *p* < 0.001) compared with excluded individuals. The prevalence of ultrasound-diagnosed hepatic steatosis was also lower among included participants (52.7% vs. 61.3%, *p* = 0.03). These differences suggest that our final cohort may represent a relatively healthier and more health-conscious segment of the screening population. This potential selection bias should be considered when interpreting the findings.

Data are expressed as mean ± SD, median (IQR), or *n* (%), with *p* values from the independent t-test, Mann–Whitney U test, or chi-square test, as appropriate. Excluded participants (*n* = 1,334) included those with baseline T2DM (*n* = 210), other liver diseases (*n* = 45), malignancy (*n* = 20), missing key baseline data (*n* = 487), loss to follow-up (*n* = 572), with 156 individuals excluded for both missing data and loss to follow-up and counted only once. Data for excluded participants were extracted from the initial screening visit where available; not all variables were available for all excluded individuals due to missing data. Percentages for excluded participants are based on available data for each variable.

### 2.3 Data collection

Data were independently extracted by two researchers from the centralized electronic database. Collected data included demographics (age, sex); anthropometrics (height, weight, BMI, waist circumference, blood pressure); laboratory parameters (fasting glucose, TG, TC, HDL-C, LDL-C, ALT, AST, GGT, and platelet count); lifestyle habits (smoking, alcohol); and medical history (hypertension). Follow-up data came from annual health examinations. All laboratory measurements followed standardized protocols at the Shanxi Bethune Hospital Institution’s central laboratory.

### 2.4 Exposure definition: MAFLD

MAFLD was diagnosed according to the 2020 international expert consensus criteria (5): hepatic steatosis on abdominal ultrasound, no other chronic liver disease, plus at least one of overweight/obesity (BMI ≥ 23 kg/m^2^), ≥2 metabolic risk abnormalities (e.g., increased waist circumference, elevated blood pressure, dyslipidemia, or prediabetes), or established T2DM (excluded at baseline). Ultrasound was performed by a team of experienced radiologists using a standardized protocol, with all examinations interpreted by the same group of readers throughout the study period to ensure consistency. Hepatic steatosis was defined by characteristic features such as increased liver echogenicity, deep beam attenuation, and vascular blurring. Although ultrasound is widely used in large-scale screening settings, it is a semi-quantitative method with inherent inter- and intra-observer variability and is less sensitive for detecting mild steatosis (<20% hepatic fat content) compared with magnetic resonance imaging-proton density fat fraction (MRI-PDFF) or controlled attenuation parameter (CAP).

### 2.5 Outcome definition

The primary outcome was new-onset T2DM during the 5-year follow-up period. T2DM was diagnosed if any of the following criteria were met during any follow-up visit: fasting plasma glucose ≥7.0 mmol/L, self-report of a physician diagnosis of T2DM with initiation of glucose-lowering therapy, or a documented diagnosis in the electronic health record. HbA1c and oral glucose tolerance testing (OGTT) were not part of the routine health check-up protocol at our center during the study period and were therefore unavailable for outcome ascertainment. Outcome assessment was standardized across all follow-up visits using the same laboratory equipment and criteria, with all fasting blood samples collected after at least 8 h of overnight fasting. Follow-up time was calculated from baseline to T2DM diagnosis, death, loss to follow-up, or study end (December 31, 2024), whichever occurred first.

All participants were scheduled to undergo annual health examinations. Participants who missed an annual visit but returned for a subsequent visit were not considered lost to follow-up; rather, their follow-up time was updated at the time of the subsequent visit. By the end of the study period, 172 participants (92.5%) had completed all scheduled annual visits. The remaining 14 participants (7.5%) had at least one missed annual visit but contributed follow-up time until their last recorded visit and were censored at that time; reasons for incomplete follow-up included relocation (*n* = 8), personal preference (*n* = 4), and unknown reasons (*n* = 2). Given that T2DM was ascertained at discrete annual health examinations rather than through continuous surveillance, interval censoring is inherently present, as the exact timing of diabetes onset between two annual visits is unknown. This interval censoring is a methodological limitation of the study design and was not addressed by the competing risk models, which only accounted for death as a competing event.

### 2.6 Statistical analysis

Statistical analyses used SPSS 26.0 and R 4.1.2. Continuous variables were compared using t-test or Mann–Whitney U test, and categorical variables using chi-square or Fisher’s exact test. Cumulative incidence of T2DM was estimated by Kaplan–Meier analysis with log-rank test. Cox regression estimated hazard ratios (HRs) and 95% CIs. Variables with *p* < 0.10 in univariate analysis entered multivariable models. The Fibrosis-4 (FIB-4) index was calculated at baseline using the following formula: FIB-4 = (age [years] × AST [U/L]) / (platelet count [10^9^/L] × √ALT [U/L]) (16). Participants were categorized into three FIB-4 risk groups based on established cut-offs: low risk (<1.30), intermediate risk (1.30–2.67), and high risk (>2.67), which have been validated in general populations for predicting advanced liver fibrosis (17). To evaluate whether the association between MAFLD and incident T2DM was independent of baseline fibrosis severity, we performed two supplementary analyses: (1) adjusting for FIB-4 as a continuous variable in the fully adjusted Cox model, and (2) adjusting for FIB-4 risk category (low, intermediate, or high) as a categorical variable. Additionally, we conducted a stratified analysis according to FIB-4 risk category to explore potential effect modification. To estimate the total effect of MAFLD on T2DM risk while avoiding overadjustment for variables that are intrinsic components of the MAFLD diagnostic criteria, we adopted a sequential modeling approach with explicit rationales: Model 1 was unadjusted (estimating the crude total effect). Model 2 was adjusted for age and sex only (estimating the effect independent of demographic factors). Model 3 was adjusted for age, sex, and BMI (estimating the effect beyond age, sex, and overall adiposity) and served as our primary adjusted model. A fully adjusted model including all covariates (age, sex, BMI, waist circumference, systolic blood pressure, TG, HDL-C, ALT) was also fitted and reported as a sensitivity analysis, but we recognize that this model may be subject to overadjustment, as several of these variables are components of the MAFLD definition; therefore, this model was used primarily for sensitivity purposes and interpreted with caution. The primary multivariable result reported throughout the abstract, methods, results, and discussion is therefore Model 3 (age-, sex-, and BMI-adjusted), with the fully adjusted model explicitly identified as a sensitivity analysis. To address the potential for overfitting, we additionally performed a sensitivity analysis using Firth’s penalized Cox regression, which is specifically designed for datasets with rare events and small sample sizes. Variables with *p* < 0.10 in univariate analysis entered multivariable models. Subgroup, stratified, and sensitivity analyses were performed, including exclusion of first-year T2DM cases, stricter T2DM definition, and competing risk analysis. For the competing risk analysis, we used the Fine-Gray subdistribution hazards model with all-cause death as the competing event, as this approach directly estimates the subdistribution hazard for T2DM in the presence of competing risks. All tests were two-sided, *p* < 0.05 significant. For stratified analyses, the number of T2DM events in each subgroup was reported to provide transparency regarding statistical power. Given the limited number of total events (*n* = 22), subgroup analyses were considered exploratory and hypothesis-generating rather than confirmatory. All interaction *p*-values were interpreted with caution, and lack of statistical significance was not interpreted as evidence of no effect modification.

To evaluate whether MAFLD adds predictive value beyond baseline dysglycemia, we conducted two additional sensitivity analyses: (1) adjusting for baseline fasting glucose as a continuous variable in the Cox model, and (2) excluding participants with baseline prediabetes (defined as fasting glucose ≥5.6 mmol/L but <7.0 mmol/L, in accordance with American Diabetes Association criteria) and repeating the primary analysis in the normoglycemic subgroup.

## 3 Results

### 3.1 Study population and baseline characteristics

A total of 1,520 individuals who underwent baseline health examinations in 2020 were initially screened. After applying the inclusion and exclusion criteria, 186 participants were eligible for the final analysis. The reasons for exclusion are detailed in Figure 1. Of the final cohort, 98 participants were classified into the MAFLD group, and 88 into the non-MAFLD group. The baseline characteristics of the study population, stratified by MAFLD status, are presented in Table 1. Compared with the non-MAFLD group, individuals in the MAFLD group were significantly older (48.5 ± 10.2 years vs. 44.3 ± 11.5 years, *p* = 0.008) and had a higher proportion of males (72.4% vs. 52.3%, *p* = 0.004). As expected, the MAFLD group exhibited a significantly worse metabolic profile, including higher BMI, waist circumference, systolic and diastolic blood pressure, fasting glucose, TG, ALT, AST, and GGT, as well as lower HDL-C levels (all *p* < 0.05). No significant intergroup differences were observed in TC, LDL-C, smoking, or alcohol consumption. The prevalence of prediabetes (fasting glucose ≥5.6 mmol/L and <7.0 mmol/L) at baseline was significantly higher in the MAFLD group compared with the non-MAFLD group: 54.1% (53/98) versus 25.0% (22/88), respectively (*p* < 0.001). The median FIB-4 index was significantly higher in the MAFLD group than in the non-MAFLD group (1.15 [IQR: 0.82–1.62] vs. 0.92 [IQR: 0.68–1.28], *p* = 0.002). According to FIB-4 risk stratification, the MAFLD group had a higher proportion of participants in the intermediate-risk category (22.4% vs. 10.2%, *p* = 0.03) and a lower proportion in the low-risk category (72.4% vs. 86.4%, *p* = 0.02); the proportion of high-risk individuals did not differ significantly between groups (5.1% vs. 3.4%, *p* = 0.56).

**Figure 1:**
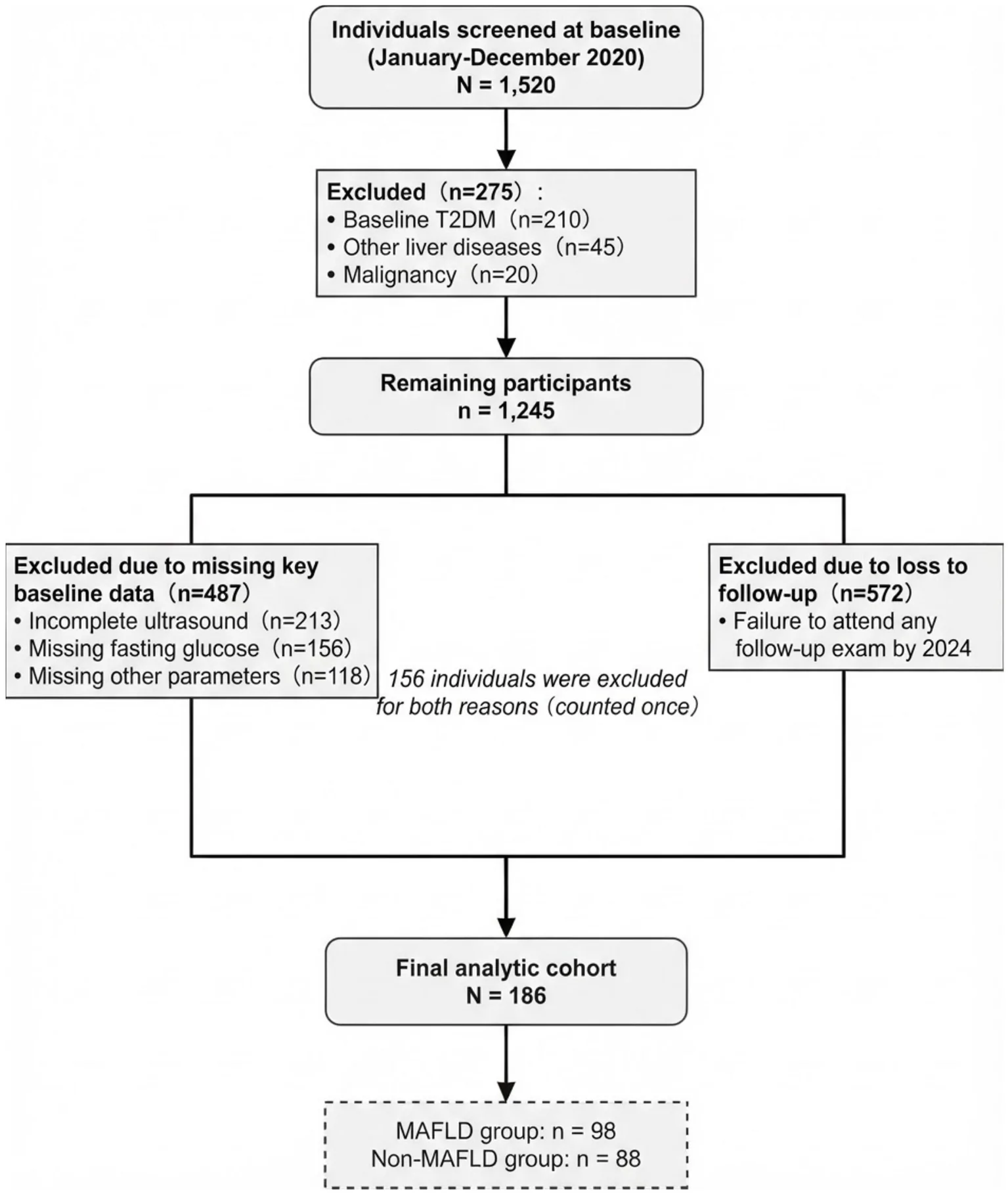
Flowchart of study participant selection. Of 1,520 individuals screened at the Shanxi Bethune Hospital Health Check-up Center (January–December 2020), after excluding those with baseline T2DM (*n* = 210), other liver diseases (*n* = 45), and malignancy (*n* = 20), 1,245 participants remained. Among these, 487 were excluded due to missing key baseline data (including incomplete ultrasound reports [*n* = 213], missing fasting glucose [*n* = 156], or missing other metabolic parameters [*n* = 118]), and 572 were excluded due to loss to follow-up (defined as failure to attend any follow-up health examination by December 31, 2024), with 156 individuals excluded for both missing data and loss to follow-up and counted only once in each category but once overall. The final cohort comprised 98 MAFLD and 88 non-MAFLD participants (total *n* = 186).

**Table 1:** Baseline characteristics of the study population stratified by MAFLD status.

Characteristic	MAFLD (*n* = 98)	Non-MAFLD (*n* = 88)	*p* value
Age, years, mean ± SD	48.5 ± 10.2	44.3 ± 11.5	0.008
Male sex, *n* (%)	71 (72.4)	46 (52.3)	0.004
BMI, kg/m^2^, mean ± SD	26.9 ± 2.9	22.8 ± 2.5	<0.001
Waist circumference, cm, mean ± SD	89.3 ± 8.7	78.5 ± 7.9	<0.001
Systolic BP, mmHg, mean ± SD	131.2 ± 12.5	122.8 ± 11.9	<0.001
Diastolic BP, mmHg, mean ± SD	83.5 ± 8.2	78.1 ± 7.5	<0.001
Fasting glucose, mmol/L, mean ± SD	5.58 ± 0.48	5.12 ± 0.43	<0.001
TG, mmol/L, median (IQR)	1.58 (1.22–2.05)	1.12 (0.85–1.48)	<0.001
TC, mmol/L, mean ± SD	4.95 ± 0.88	4.78 ± 0.85	0.179
HDL-C, mmol/L, mean ± SD	1.12 ± 0.25	1.35 ± 0.28	<0.001
LDL-C, mmol/L, mean ± SD	3.05 ± 0.75	2.95 ± 0.72	0.353
ALT, U/L, median (IQR)	32.0 (25.0–42.0)	22.0 (18.0–28.0)	<0.001
AST, U/L, median (IQR)	25.0 (21.0–31.0)	20.0 (17.0–24.0)	<0.001
GGT, U/L, median (IQR)	38.5 (28.0–52.0)	24.0 (18.0–32.0)	<0.001
Current smoker, *n* (%)	28 (28.6)	20 (22.7)	0.357
Alcohol consumption, *n* (%)	35 (35.7)	25 (28.4)	0.280
Hypertension, *n* (%)	42 (42.9)	19 (21.6)	0.002

Data are expressed as mean ± SD, median (IQR), or *n* (%), with *p* values from the independent *t*-test, Mann–Whitney U test, or chi-square test, as appropriate. ALT, alanine aminotransferase; AST, aspartate aminotransferase; BMI, body mass index; BP, blood pressure; GGT, gamma-glutamyl transferase; HDL-C, high-density lipoprotein cholesterol; IQR, interquartile range; LDL-C, low-density lipoprotein cholesterol; MAFLD, metabolic dysfunction-associated fatty liver disease; SD, standard deviation; TC, total cholesterol; TG, triglycerides.

A comparison of baseline characteristics between included (*n* = 186) and excluded (*n* = 1,334) participants is provided in Supplementary Table S1. Included participants were significantly younger, had lower BMI, lower fasting glucose, lower ALT levels, and a lower prevalence of hepatic steatosis compared with excluded individuals (all *p* < 0.05, with full details in Supplementary Table S1). These differences suggest that the final analytic cohort may be somewhat healthier than the overall screened population, which should be considered when generalizing the findings.

### 3.2 Incidence of new-onset T2DM

Over a median follow-up period of 60 months (range: 12–60 months), 22 participants (11.8%) developed new-onset T2DM. A total of 172 participants (92.5%) completed all scheduled annual visits through the end of follow-up, while 14 participants (7.5%) were censored before the study end due to missed visits. The MAFLD group had a significantly higher number of new T2DM cases (*n* = 18, 18.4%) compared to the non-MAFLD group (*n* = 4, 4.5%). The 5-year cumulative incidence of T2DM was 18.9% in the MAFLD group and 5.6% in the non-MAFLD group (*p* = 0.001). The incidence density of T2DM was 38.5 per 1,000 person-years in the MAFLD group, compared to 9.5 per 1,000 person-years in the non-MAFLD group (*p* < 0.001). These findings are summarized in Table 2.

**Table 2:** Comparison of new-onset T2DM during follow-up.

Outcome	MAFLD (*n* = 98)	Non-MAFLD (*n* = 88)	*p* value
New-onset T2DM, *n* (%)	18 (18.4)	4 (4.5)	0.003
5-year cumulative incidence, %	18.9	5.6	0.001
Incidence density (per 1,000 person-years)	38.5	9.5	<0.001

### 3.3 Association between baseline MAFLD and risk of T2DM

The Kaplan–Meier survival curve (Figure 2) demonstrated that participants with MAFLD had a significantly lower probability of remaining free from T2DM during the 5-year follow-up compared to those without MAFLD (log-rank *p* < 0.001). Cox regression results are shown in Table 3. MAFLD was associated with increased T2DM risk in unadjusted (HR = 4.28, 95%CI: 2.08–8.81, *p* < 0.001), age- and sex-adjusted (HR = 3.62, 95%CI: 1.82–7.20, *p* < 0.001), and the primary age-, sex-, and BMI-adjusted model (HR = 3.15, 95%CI: 1.58–6.28, *p* = 0.001). The fully adjusted sensitivity model (including additional metabolic covariates) yielded consistent results (HR = 2.71, 95%CI: 1.33–5.52, *p* = 0.006). To evaluate whether the observed association was independent of baseline liver fibrosis severity, we further adjusted for the FIB-4 index. When FIB-4 was added as a continuous covariate to the fully adjusted model, the association between MAFLD and incident T2DM remained significant (HR = 2.45, 95%CI: 1.18–5.09, *p* = 0.02). Similarly, when FIB-4 risk category (low, intermediate, or high) was included as a categorical covariate, the HR for MAFLD was 2.38 (95%CI: 1.14–4.97, *p* = 0.02). These findings suggest that the association between MAFLD and T2DM is independent of baseline fibrosis risk, although the limited number of events in the high-risk subgroup (*n* = 8, with 2 T2DM events) warrants cautious interpretation. To further address the concern of model overfitting due to the limited number of events, we performed sensitivity analyses using Firth’s penalized Cox regression. The results remained generally consistent with the primary analysis (Firth-corrected HR for MAFLD: 2.58, 95%CI: 1.28–5.21, *p* = 0.008), providing additional support for the primary association, although these findings remain preliminary given the small sample size.

**Figure 2:**
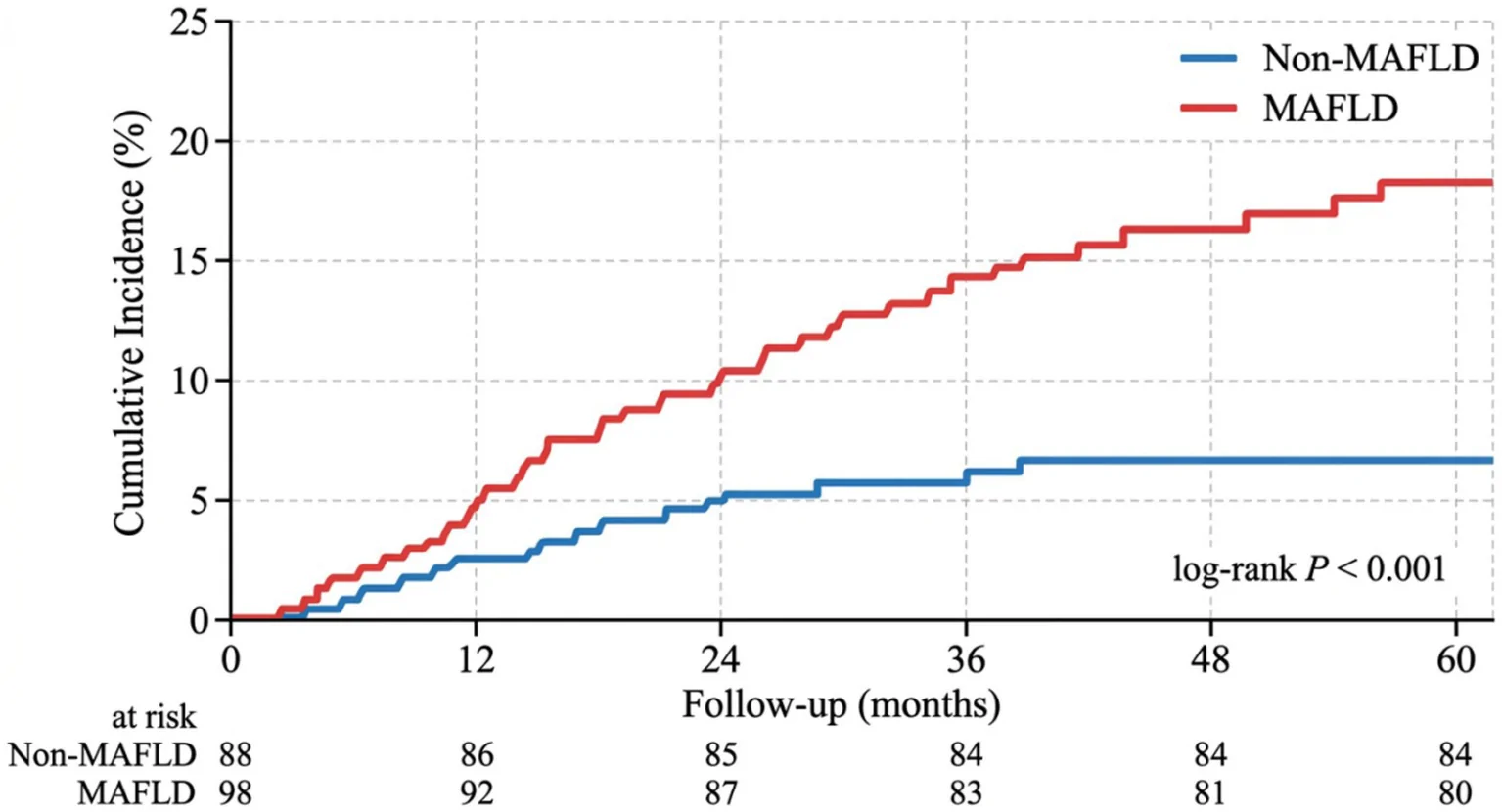
Kaplan–Meier curve for cumulative incidence of new-onset T2DM, stratified by baseline MAFLD status (non-MAFLD: blue, *n* = 88; MAFLD: red, *n* = 98). The MAFLD group had significantly higher cumulative incidence over 5 years (log-rank *p* < 0.001). Numbers at risk at 0, 12, 24, 36, 48, and 60 months: non-MAFLD: 88, 86, 85, 84, 84, 84; MAFLD: 98, 92, 87, 83, 81, 80.

**Table 3:** Cox proportional hazards regression analysis for the association between MAFLD and new-onset T2DM.

Model	HR (95% CI)	*p* value
Model 1 (Unadjusted)	4.28 (2.08–8.81)	<0.001
Model 2 (Adjusted for age, sex)	3.62 (1.82–7.20)	<0.001
Model 3 (Primary model: adjusted for age, sex, and BMI)	3.15 (1.58–6.28)	0.001

Model 3, adjusted for age, sex, and BMI, served as the primary multivariable model. Given the limited number of outcome events (*n* = 22), this model was interpreted with caution. The primary inference was based on Model 3. A fully adjusted sensitivity model including additional metabolic covariates (waist circumference, systolic blood pressure, triglycerides, HDL-C, and ALT) was also performed and yielded consistent results (HR = 2.71, 95% CI: 1.33–5.52, *p* = 0.006), although this model may be subject to overadjustment and was therefore interpreted with caution. All subgroup analyses by MAFLD subtype (Table 4) were derived from this fully adjusted sensitivity model.

**Table 4:** Risk of new-onset T2DM by MAFLD subtype.

Subtype	*n*	HR (95% CI)*	*p* value
Non-MAFLD	88	1.00 (Reference)	-
Lean MAFLD (BMI < 23 kg/m^2^)	15	2.88 (1.10–7.54)	0.03
Overweight/Obese MAFLD (BMI ≥ 23 kg/m^2^)	83	2.65 (1.22–5.76)	0.01

*p < 0.05.

### 3.4 Risk of T2DM by MAFLD subtypes

Among the 18 T2DM events that occurred in the MAFLD group, 3 events (20.0%) were observed in the lean MAFLD subgroup (*n* = 15), and 15 events (18.1%) in the overweight/obese MAFLD subgroup (*n* = 83). Of the 98 participants with MAFLD, 15 (15.3%) were classified as having lean MAFLD (BMI < 23 kg/m^2^), and 83 (84.7%) as having overweight/obese MAFLD (BMI ≥ 23 kg/m^2^). Among these, 3 T2DM events occurred in the lean MAFLD subgroup and 15 events in the overweight/obese MAFLD subgroup. The forest plot (Figure 3) and Cox regression analysis (Table 4) showed that, compared to the non-MAFLD reference group, both lean MAFLD (HR = 2.88, 95%CI: 1.10–7.54, *p* = 0.03) and overweight/obese MAFLD (HR = 2.65, 95%CI: 1.22–5.76, *p* = 0.01) were suggestive of an increased risk of new-onset T2DM in the fully adjusted sensitivity models. However, given the very small number of participants (*n* = 15) and events (*n* = 3) in the lean MAFLD subgroup, this analysis was severely underpowered and the finding should be considered exploratory only, requiring confirmation in larger cohorts.

**Figure 3:**
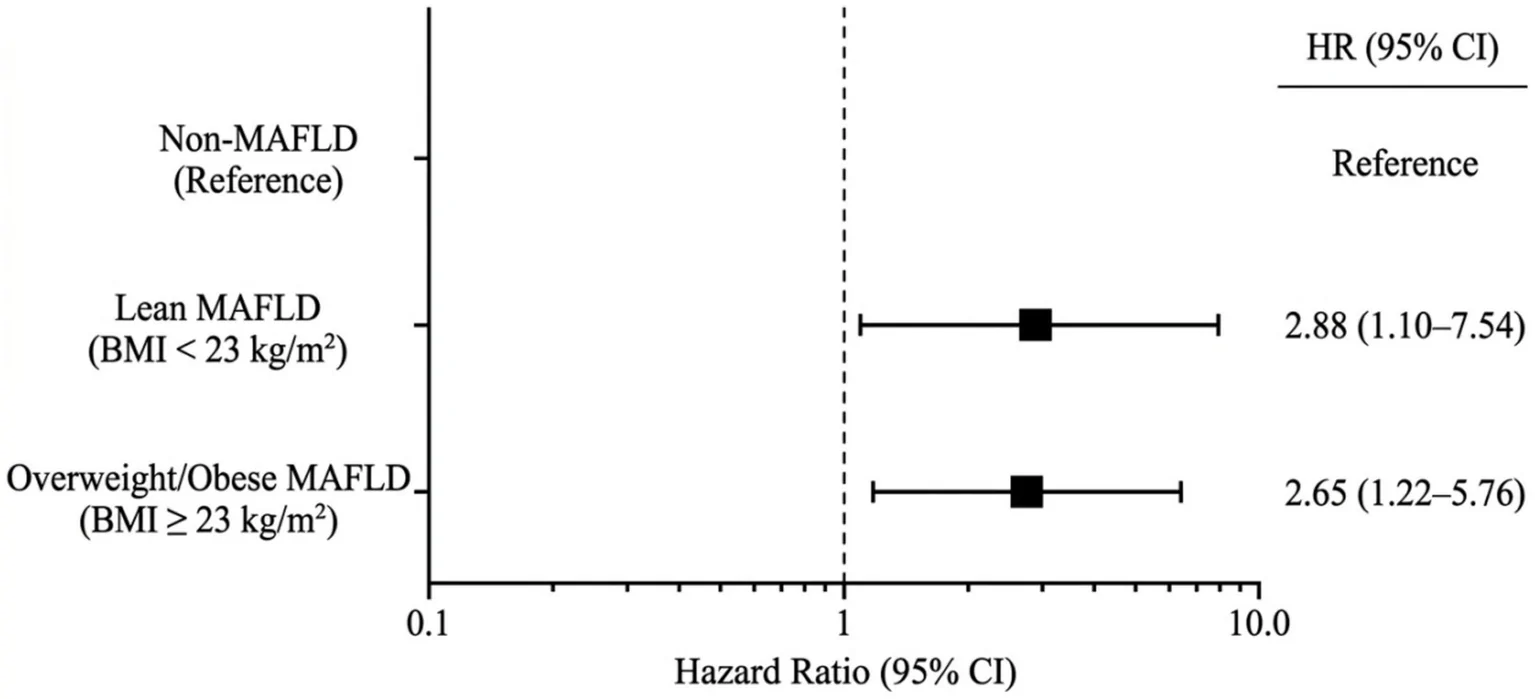
Forest plot of MAFLD subtypes and risk of new-onset T2DM. Compared to the non-MAFLD group, lean MAFLD (BMI < 23 kg/m^2^; HR 2.88, 95% CI 1.10–7.54, *p* = 0.03) and overweight/obese MAFLD (BMI ≥ 23 kg/m^2^; HR 2.65, 95% CI 1.22–5.76, *p* = 0.01) showed significantly elevated risks. HRs were adjusted for age, sex, waist circumference, systolic blood pressure, triglycerides, HDL-C, and ALT.

### 3.5 Stratified and sensitivity analyses

Stratified analyses were performed to explore the consistency of the association between MAFLD and T2DM across different subgroups (Figure 4). The number of T2DM events in each subgroup was as follows: males, 15 events; females, 7 events; age <50 years, 9 events; age ≥50 years, 13 events; BMI < 24 kg/m^2^, 8 events; BMI ≥ 24 kg/m^2^, 14 events. The increased risk associated with MAFLD was suggested in males (HR = 2.58, 95%CI: 1.18–5.64), females (HR = 2.95, 95%CI: 1.02–8.53), individuals aged <50 years (HR = 3.12, 95%CI: 1.28–7.61), individuals aged ≥50 years (HR = 2.43, 95%CI: 1.08–5.47), those with BMI < 24 kg/m^2^ (HR = 3.05, 95%CI: 1.21–7.69), and those with BMI ≥ 24 kg/m^2^ (HR = 2.48, 95%CI: 1.10–5.59). None of the interaction terms were statistically significant (all *P* for interaction >0.05). However, given the small number of events in each subgroup, these stratified analyses were underpowered and should be interpreted as exploratory only; the lack of significant interaction should not be interpreted as definitive evidence of no effect modification.

**Figure 4:**
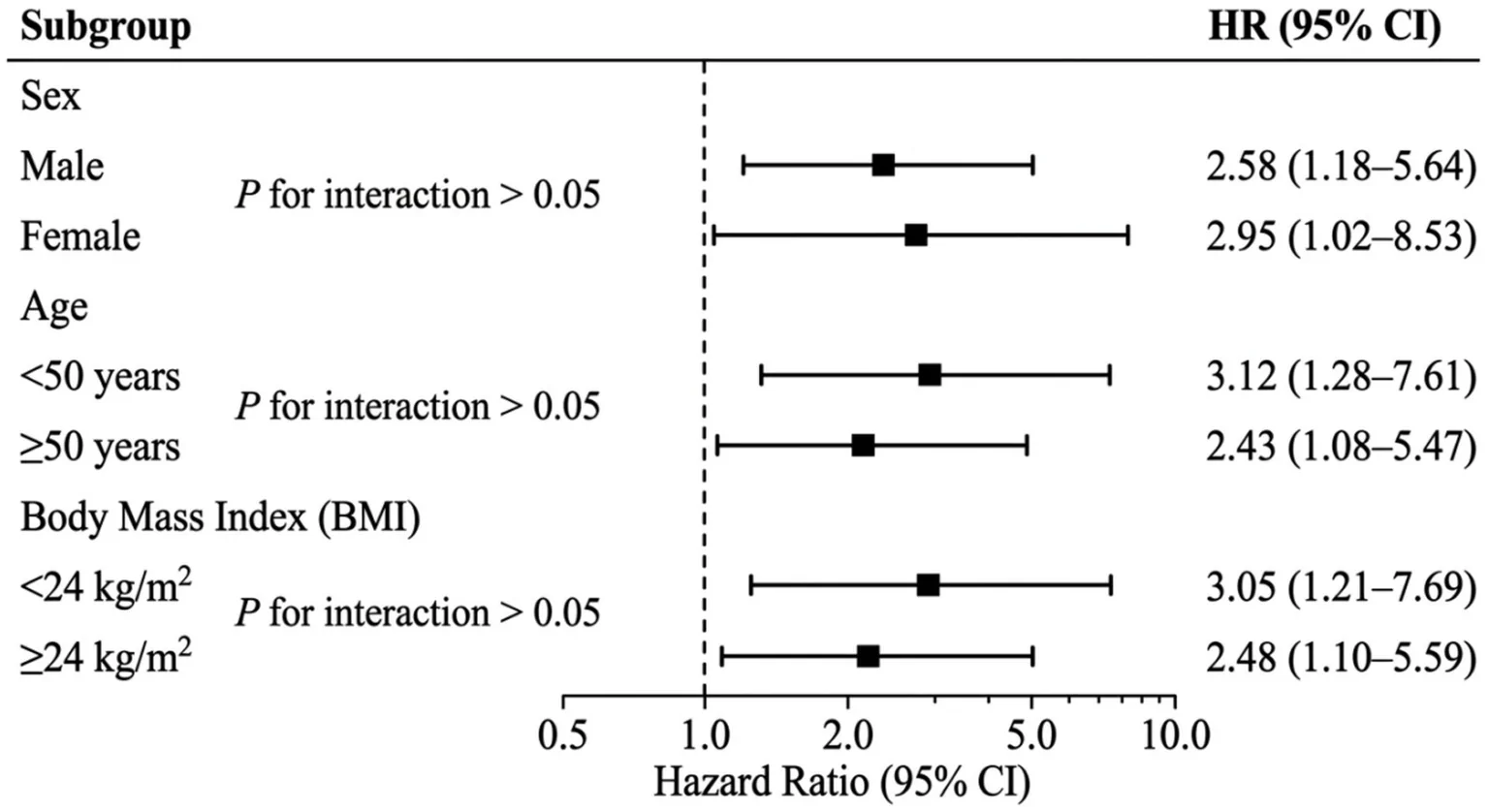
Forest plot of stratified analyses for MAFLD and new-onset T2DM. The number of T2DM events in each subgroup is shown in parentheses. Point estimates suggested an association between MAFLD and T2DM across all subgroups (HR range 2.43–3.12), but confidence intervals were wide, and all subgroup analyses were underpowered due to the small number of events. No significant interaction was detected (all interaction *p* > 0.05), but the lack of significance should not be interpreted as evidence of no effect modification. Subgroups included sex, age (<50 vs. ≥50 years), and BMI (<24 vs. ≥24 kg/m^2^). HRs were adjusted for age, sex, and BMI as appropriate for each subgroup.

The results of the sensitivity analyses are presented in Table 5. After excluding 3 participants who developed T2DM within the first year of follow-up, the association remained robust (HR = 2.52, 95%CI: 1.18–5.38, *p* = 0.02). Using a competing risk model that accounted for all-cause death as a competing event using the Fine-Gray subdistribution hazards approach did not materially alter the findings (HR = 2.65, 95%CI: 1.28–5.49, *p* = 0.009). During the 5-year follow-up, 2 deaths (1.1%) were recorded (one in the MAFLD group and one in the non-MAFLD group), both due to causes unrelated to T2DM; the small number of competing events limited the statistical power of this analysis, and the results should be interpreted cautiously. Finally, when a stricter definition of T2DM (fasting plasma glucose ≥7.0 mmol/L) was applied, MAFLD remained a significant predictor (HR = 2.58, 95%CI: 1.20–5.55, *p* = 0.02). These sensitivity analyses were generally consistent with and supported the primary findings, although the small number of events warrants cautious interpretation.

**Table 5:** Sensitivity analyses for the association between MAFLD and new-onset T2DM.

Sensitivity analysis	HR (95% CI)*	*p* value
Excluding T2DM cases within first year	2.52 (1.18–5.38)	0.02
Competing risk model (death as competing event)	2.65 (1.28–5.49)	0.009
Stricter T2DM definition (FPG ≥ 7.0 mmol/L)	2.58 (1.20–5.55)	0.02

*p < 0.05.

To assess whether MAFLD confers risk beyond baseline dysglycemia, we performed two additional sensitivity analyses. First, after additional adjustment for baseline fasting glucose as a continuous variable, the association between MAFLD and incident T2DM remained significant but was attenuated (HR = 1.98, 95%CI: 1.02–3.84, *p* = 0.04). Second, when we excluded 75 participants with baseline prediabetes and repeated the analysis in the normoglycemic subgroup (*n* = 111), MAFLD remained associated with an increased risk of T2DM (HR = 3.15, 95%CI: 1.18–8.42, *p* = 0.02), with 5 events in the MAFLD group and 2 events in the non-MAFLD group. These findings suggest that MAFLD might add predictive value for T2DM even beyond baseline glucose status, although the small number of events in the normoglycemic subgroup warrants cautious interpretation and this observation should be considered exploratory.

In a supplementary stratified analysis according to baseline FIB-4 risk category, the association between MAFLD and incident T2DM appeared consistent across FIB-4 strata: among participants with low FIB-4 (<1.30, *n* = 148, 14 events), the adjusted HR for MAFLD was 2.85 (95%CI: 1.21–6.71, *p* = 0.02); among those with intermediate FIB-4 (1.30–2.67, *n* = 30, 6 events), the adjusted HR was 2.42 (95%CI: 0.88–6.65, *p* = 0.09); and among those with high FIB-4 (>2.67, *n* = 8, 2 events), the adjusted HR was 2.15 (95%CI: 0.45–10.28, *p* = 0.34). However, the small number of participants and events in the intermediate and high FIB-4 categories substantially limited statistical power, and the interaction term between MAFLD and FIB-4 category was not statistically significant (*P* for interaction = 0.68). These subgroup findings should therefore be considered exploratory and hypothesis-generating only.

## 4 Discussion

This retrospective cohort study, conducted within a real-world health check-up population at a single center in China, provides preliminary evidence suggesting that MAFLD, diagnosed according to the 2020 consensus criteria, may be associated with an increased risk of the development of T2DM over a 5-year period. However, given the single-center design, small sample size, and high exclusion rate, these findings should be considered hypothesis-generating and require validation in larger, more diverse, and prospectively designed cohorts. The key findings suggest that individuals with MAFLD had a 3.15-fold higher risk of incident T2DM after adjusting for age, sex, and BMI in the primary analysis (HR = 3.15; a fully adjusted sensitivity analysis yielded HR = 2.71). Notably, this elevated risk was suggested not only in the well-recognized overweight/obese MAFLD subgroup but also preliminarily in individuals with lean MAFLD, although the latter finding is based on a very small sample (*n* = 15, 3 events) and should be interpreted with particular caution. The overall pattern of these findings was broadly supported by results across multiple stratified and sensitivity analyses, although the limited statistical power precludes definitive conclusions. Collectively, these data underscore the critical role of MAFLD as a high-risk state for T2DM and highlight the importance of its identification in routine clinical practice.

The core innovation of this study lies in its application of the contemporary MAFLD definition within a typical health check-up setting, providing validation of its predictive utility for T2DM in this specific population. While a substantial body of literature has established a link between NAFLD and incident diabetes (9, 18), the transition to the MAFLD paradigm, which inherently captures the metabolic dysfunction component, provides a more precise and clinically relevant characterization of at-risk individuals. Our findings are consistent with a recent meta-analysis of 33 studies, which reported a pooled HR of 2.19 for the association between NAFLD and T2DM (9). However, our study extends these observations by demonstrating that the MAFLD definition, with its requirement for metabolic comorbidity, identifies a population with a similarly elevated risk, validating its use for risk prediction. Furthermore, our study’s exclusive focus on a community-dwelling health check-up cohort, as opposed to hospital-based populations common in prior research (10), may reduce some sources of selection bias. Nonetheless, the generalizability of our findings to the broader population of asymptomatic individuals undergoing routine screening is constrained by the single-center design, the relatively small sample size, and the high proportion of excluded participants, which may have introduced selection bias. Therefore, our results should be viewed as preliminary and in need of replication in larger, multi-center cohorts. This setting represents a useful complement to prior validation studies for MAFLD’s prognostic value, many of which were conducted in hepatology or endocrine clinics, where referral bias may have inflated the observed associations. However, given the exploratory nature of our findings, this should not be overinterpreted as a definitive advancement (19).

A particularly preliminary finding of our study is the suggested association between lean MAFLD and incident T2DM. This observation, based on a very small subgroup of only 15 lean MAFLD participants and only 3 T2DM events, must be interpreted with extreme caution and should be considered exploratory. It provides a hypothesis-generating indication that metabolic dysfunction, rather than excess adiposity per se, might be a key driver of diabetes risk, but this finding is severely underpowered and should not be overinterpreted. Given the limited sample size, this finding should be viewed as exploratory and hypothesis-generating only, and larger prospective studies are urgently needed to investigate the risk associated with lean MAFLD. Lean MAFLD represents a unique phenotype, often characterized by a higher degree of genetic susceptibility and a more pronounced hepatic insulin resistance despite normal BMI (20). A systematic review and meta-analysis reported that lean individuals with NAFLD had a 2.5-fold increased risk of incident T2DM compared to lean individuals without NAFLD (13). Our findings align with this evidence and extend it by using the MAFLD framework, demonstrating that lean individuals who meet the metabolic criteria for MAFLD are at a significant and comparable risk to their overweight/obese counterparts. This has profound clinical implications, as lean individuals with MAFLD are often overlooked in routine screening and may not receive the same level of preventative counseling as their overweight/obese peers (21). The comparable risk between lean and overweight/obese MAFLD subgroups in our study suggests that the metabolic dysfunction component, rather than adiposity itself, is the primary driver of the heightened diabetes risk.

The link between MAFLD and T2DM involves multifactorial mechanisms centered on the liver as a key site of insulin resistance (22). Intrahepatic diacylglycerol accumulation activates protein kinase C epsilon, impairing insulin signaling and increasing gluconeogenesis (8). The steatotic liver secretes hepatokines (e.g., fetuin-A, FGF-21) that affect peripheral insulin sensitivity (23) and releases pro-inflammatory cytokines (e.g., IL-6, TNF-*α*) that promote systemic low-grade inflammation (24). This interplay of hepatic insulin resistance, lipotoxicity, and chronic inflammation impairs pancreatic beta-cell function, creating a pro-diabetic milieu that explains the elevated risk even in lean MAFLD individuals (25). An important additional finding from our study is that the association between MAFLD and incident T2DM remained significant after adjustment for the FIB-4 index, a non-invasive marker of liver fibrosis severity (HR = 2.45, 95%CI: 1.18–5.09). This suggests that the elevated diabetes risk conferred by MAFLD is not merely a reflection of underlying advanced fibrosis, but rather appears to be driven by the metabolic dysfunction component of the disease. This observation is consistent with prior studies demonstrating that insulin resistance and hepatic steatosis per se, rather than fibrosis stage, are the primary drivers of impaired glucose metabolism in fatty liver disease (26). However, given the small number of participants with intermediate or high FIB-4 scores in our cohort (*n* = 38 combined), these findings should be interpreted cautiously and require validation in larger cohorts with more detailed fibrosis assessment, such as transient elastography or magnetic resonance elastography.

If confirmed in future larger studies, the clinical implications of our findings could be relevant to health management. First, our study provides preliminary support for the view that a diagnosis of MAFLD may help identify individuals who could be at potentially high risk for T2DM, although this clearly requires further prospective validation in larger cohorts. Should these findings be replicated, this risk assessment might prompt more intensive and regular screening for dysglycemia, including annual measurement of fasting glucose and possibly glycated hemoglobin (HbA1c), in this population (27). Second, the identification of MAFLD provides a crucial opportunity for early intervention. Lifestyle modification, primarily focused on weight loss (5–10% reduction), has been proven to effectively reduce liver fat content and improve insulin sensitivity (28). For patients with lean MAFLD, the focus should shift toward improving body composition through increased physical activity and dietary quality, rather than weight loss alone (29). Third, from a public health perspective, incorporating MAFLD status into the risk stratification algorithms of health check-up programs could enable a more targeted and cost-effective approach to diabetes prevention. This could involve providing enhanced lifestyle counseling and more frequent follow-up for individuals identified with MAFLD (30). The simplicity of MAFLD diagnosis using routine ultrasound and metabolic parameters makes it an ideal tool for implementation in primary care and health screening settings.

This study has several limitations. First and foremost, the small sample size (*n* = 186) and limited number of outcome events (*n* = 22) substantially reduce statistical power, particularly for subgroup analyses, and preclude any claim of robust or definitive inference. All findings, including the primary association, should therefore be viewed as preliminary and hypothesis-generating rather than confirmatory. First, its retrospective, single-center design and modest sample size (*n* = 186), with only 15 participants (15.3%) in the lean MAFLD subgroup, substantially limit statistical power and preclude robust subgroup analyses. Consequently, the observed association between lean MAFLD and incident T2DM (HR = 2.88) should be interpreted as a preliminary indication that requires confirmation in larger, adequately powered prospective cohorts. Second, hepatic steatosis was diagnosed by ultrasound rather than the gold-standard liver biopsy or MRI-PDFF. Although all ultrasound examinations were performed and interpreted by a consistent team of experienced radiologists, ultrasound remains a semi-quantitative tool with operator-dependent variability and limited sensitivity for mild steatosis, which may lead to misclassification of exposure. Nonetheless, ultrasound remains the most practical and cost-effective imaging modality for routine health screening in large populations. Third, data on lifestyle factors (e.g., dietary habits, physical activity levels) were unavailable in this retrospective database. Moreover, T2DM diagnosis was based solely on fasting plasma glucose (≥7.0 mmol/L) and medical records, without incorporating oral glucose tolerance test (OGTT) or glycated hemoglobin (HbA1c) criteria. This approach may have underestimated the true incidence of T2DM, as OGTT is more sensitive for detecting postprandial hyperglycemia and early insulin resistance, particularly in individuals with isolated impaired glucose tolerance. Future prospective studies should incorporate OGTT and HbA1c measurements at baseline and during follow-up to capture a more comprehensive spectrum of glucose metabolism abnormalities. Fourth, lack of external validation limits generalizability. Future research should focus on validating these results in diverse populations across different geographic regions and ethnic groups to confirm the external validity of our observations. In addition, future studies should incorporate more objective and quantitative tools for hepatic steatosis assessment, such as MRI-PDFF, FibroScan with controlled attenuation parameter (CAP), or non-invasive fibrosis scores (e.g., FIB-4 or NFS), to reduce misclassification bias and enable more precise risk stratification. In summary, while our findings are suggestive of an association between MAFLD and incident T2DM and are supported by biological plausibility, the methodological limitations inherent to this single-center retrospective study-particularly the small sample size, limited number of outcome events, high exclusion rate, the inherent interval censoring due to annual outcome assessment (whereby the exact timing of T2DM onset between visits is unknown), and lack of external validation-necessitate a very cautious interpretation. The results should be viewed as exploratory and hypothesis-generating and requiring confirmation in larger, multi-center prospective studies.

## 5 Conclusion

In conclusion, this retrospective cohort study provides preliminary evidence suggesting that baseline MAFLD may be associated with an increased risk of new-onset T2DM within five years in a health check-up population. However, due to the single-center design, small sample size, and limited number of outcome events, these findings should be interpreted as exploratory and preliminary and hypothesis-generating. Larger, multi-center prospective studies with more comprehensive outcome ascertainment are needed to confirm these observations and to establish the generalizability of the findings. The suggested association was observed preliminarily in both lean and overweight/obese MAFLD subtypes, although the lean MAFLD finding is based on a very small sample (*n* = 15, 3 events) and should be interpreted as an exploratory observation only that warrants confirmation in larger validation cohorts. If confirmed in future studies, these findings could underscore the importance of recognizing MAFLD, diagnosed by the 2020 consensus criteria, as a potential risk marker for T2DM in routine clinical practice. However, given the exploratory and preliminary nature of our results, any clinical or public health implications should be considered speculative at this stage. Pending such validation, proactive screening for MAFLD in health check-up settings and the implementation of targeted lifestyle interventions for affected individuals might represent a potential strategy for the primary prevention of diabetes, but this requires confirmation in adequately powered prospective studies before any practice recommendations can be made.

## Data Availability

The original contributions presented in the study are included in the article/Supplementary material, further inquiries can be directed to the corresponding author.
