# Supplementary material for: Metabolic dysfunction-associated fatty liver disease increases the risk of new-onset type 2 diabetes mellitus: a retrospective cohort study based on health check-up data
Source: Front Med (Lausanne). 2026 Sep 16;13:1881173. doi: 10.3389/fmed.2026.1881173 (PMC13623719; doi:10.3389/fmed.2026.1881173)
Supplement: Supplementary file 1 [file Table_1.docx]

**Supplementary Table S1.** Comparison of baseline characteristics between included and excluded participants

| ****Characteristic**** | ****Included (n=186)**** | ****Excluded (n=1,334)**** | *****P* value**** |
| --- | --- | --- | --- |
| **Age, years, mean ± SD** | **46.5 ± 10.9** | **51.2 ± 12.3** | ***<0.001*** |
| **Male sex, n (%)** | **117 (62.9)** | **801 (60.0)** | ***0.45*** |
| **BMI, kg/m², mean ± SD** | **24.9 ± 3.2** | **26.1 ± 3.5** | ***<0.001*** |
| **Waist circumference, cm, mean ± SD** | **83.9 ± 9.5** | **86.4 ± 10.2** | ***0.002*** |
| **Systolic BP, mmHg, mean ± SD** | **127.0 ± 12.8** | **129.5 ± 13.5** | ***0.02*** |
| **Diastolic BP, mmHg, mean ± SD** | **80.8 ± 8.1** | **82.1 ± 8.5** | ***0.06*** |
| **Fasting glucose, mmol/L, mean ± SD** | **5.35 ± 0.48** | **5.58 ± 0.62** | ***<0.001*** |
| **TG, mmol/L, median (IQR)** | **1.35 (0.98-1.82)** | **1.52 (1.08-2.10)** | ***<0.001*** |
| **TC, mmol/L, mean ± SD** | **4.87 ± 0.87** | **4.92 ± 0.90** | ***0.48*** |
| **HDL-C, mmol/L, mean ± SD** | **1.23 ± 0.28** | **1.18 ± 0.30** | ***0.04*** |
| **LDL-C, mmol/L, mean ± SD** | **3.00 ± 0.74** | **3.04 ± 0.76** | ***0.50*** |
| **ALT, U/L, median (IQR)** | **26.0 (19.0-35.0)** | **30.0 (22.0-42.0)** | ***<0.001*** |
| **AST, U/L, median (IQR)** | **22.0 (18.0-28.0)** | **24.0 (19.0-31.0)** | ***<0.001*** |
| **GGT, U/L, median (IQR)** | **30.0 (22.0-42.0)** | **34.0 (25.0-48.0)** | ***<0.001*** |
| **Hepatic steatosis on ultrasound, n (%)** | **98 (52.7)** | **818 (61.3)** | ***0.03*** |
| **Current smoker, n (%)** | **48 (25.8)** | **335 (25.1)** | ***0.84*** |
| **Alcohol consumption, n (%)** | **60 (32.3)** | **398 (29.8)** | ***0.50*** |
| **Hypertension, n (%)** | **61 (32.8)** | **498 (37.3)** | ***0.23*** |

Data are expressed as mean ± SD, median (IQR), or n (%), with *P* values from the independent t-test, Mann-Whitney U test, or chi-square test, as appropriate. Excluded participants (n=1,334) included those with baseline T2DM (n=210), other liver diseases (n=45), malignancy (n=20), missing key baseline data (n=487), loss to follow-up (n=572), with 156 individuals excluded for both missing data and loss to follow-up and counted only once. Data for excluded participants were extracted from the initial screening visit where available; not all variables were available for all excluded individuals due to missing data. Percentages for excluded participants are based on available data for each variable.
